# Excessive Stretching Drives RPE Inflammation and ECM Remodeling in Ectopia Lentis Retinopathy

**DOI:** 10.3390/ijms27114870

**Published:** 2026-05-28

**Authors:** Yan Liu, Zijia Zhao, Linghao Song, Xinyue Wang, Yinuo Wen, Shenjie Peng, Min Zhang, Zexu Chen, Tianhui Chen, Yongxiang Jiang

**Affiliations:** 1 Eye Institute and Department of Ophthalmology, Eye & ENT Hospital, Fudan University, Shanghai 200031, China; liuyan218821@163.com (Y.L.); zjzhao97@gmail.com (Z.Z.); songlinghao333@163.com (L.S.); 18781715124@163.com (X.W.); 25111260024@m.fudan.edu.cn (Y.W.); zhang_m13@fudan.edu.cn (M.Z.); chenzexu430@163.com (Z.C.); 2 NHC Key Laboratory of Myopia (Fudan University), Key Laboratory of Myopia, Chinese Academy of Medical Sciences, Shanghai 200031, China; 3 Shanghai Key Laboratory of Visual Impairment and Restoration, Shanghai 200031, China; 4 Shanghai Medical College (SHMC), Fudan University, Shanghai 200031, China; jspeng5623@163.com

**Keywords:** ectopia lentis, axial elongation, retinal pigment epithelial cells, cyclic mechanical stretch, RNA sequencing

## Abstract

This study investigated the posterior segment manifestations and molecular mechanisms of retinopathy in ectopia lentis (EL) by subjecting retinal pigment epithelial (RPE) cells to excessive mechanical stretching. A total of 127 patients with EL and 149 healthy controls underwent comprehensive ophthalmic examinations. Aqueous humor from 10 patients per group underwent untargeted metabolomic analysis. In vitro, ARPE-19 cells were subjected to excessive mechanical stretching using a cell-tank device mimicking axial elongation. The results showed that patients with EL exhibited significantly longer Z-AL than controls, with 36.22% demonstrating retinal abnormalities. Metabolomic and RNA-seq analyses revealed enriched inflammatory metabolites and activated ECM-remodeling pathways. Real-time quantitative PCR (RT-qPCR), immunofluorescence (IF), Western blotting (WB), and an enzyme-linked immunosorbent assay (ELISA) confirmed elevated secretion of TNF-α, IL-6, and MMP3 in stretched RPE cells, consistent with the metabolomic findings. Excessive mechanical stretching induces inflammatory and ECM remodeling responses in RPE cells, potentially contributing to the retinopathy observed in EL. By integrating clinical, metabolomic, and transcriptomic data, this study highlights TNF-α, IL-6, and MMP3 as inflammatory mediators linking biomechanical stress to retinal abnormalities. These findings provide insights into disease-associated retinal remodeling in EL and may inform future therapeutic strategies.

## 1. Introduction

Congenital ectopia lentis (EL) is a rare inherited ocular disorder characterized by displacement of the crystalline lens from its normal anatomical position due to defective zonular fibers [1]. Patients with EL commonly exhibit progressive axial elongation, resulting in high myopia and associated complications such as posterior staphyloma, macular pathology, and retinal detachment (RD) [2]. The incidence of RD ranges from 5% to 11% and may reach as high as 69% in bilateral cases [3]. Both partial and complete ectopia lentis can exert traction on the vitreous base, potentially inducing peripheral retinal holes or tears [4,5]. In addition, axial elongation promotes degenerative changes, such as early vitreous liquefaction and detachment, retinal thinning, lattice degeneration, and peripheral retinal breaks, all of which increase the risk of multiple large or even giant retinal tears in EL patients [6].

**Figure 1:**
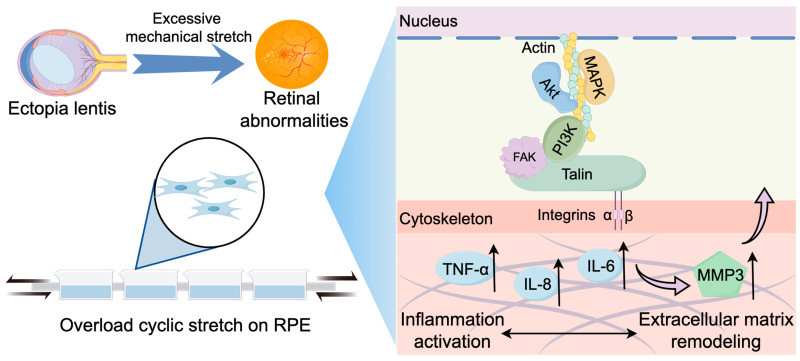
Schematic diagram illustrating the pathogenic mechanisms of retinal alterations associated with ectopia lentis (EL). This illustration outlines the key pathological mechanisms linking EL to retinal abnormalities. Mechanical stretching in EL eyes activates inflammatory responses, which in turn induce extracellular matrix remodeling involving actin cytoskeleton, FAK, and talin. FAK, focal adhesion kinase; TNF-α, tumor necrosis factor-α; IL-6, interleukin-6; MMP3, matrix metalloproteinase-3.

Despite the well-recognized clinical risks, the biomechanical and cellular responses of retinal pigment epithelial (RPE) cells to mechanical stretching, which simulates axial elongation in EL, remain poorly understood [7,8,9]. RPE cells are highly sensitive to mechanical cues and play a pivotal role in maintaining retinal homeostasis. Therefore, understanding their mechanobiological responses may provide critical insights into the pathogenesis of EL-related vitreoretinal complications (Figure 1).

In this study, we established an in vitro overloaded cyclic stretching model using ARPE-19 cells to mimic the mechanical strain experienced by the retina during axial elongation [7,8,9,10,11]. In parallel, untargeted metabolomic profiling of aqueous humor obtained from EL patients revealed metabolic signatures related to inflammation. Furthermore, transcriptomic analysis of stretched ARPE-19 cells confirmed activation of inflammatory pathways and upregulation of extracellular matrix (ECM)-remodeling genes [12,13,14].

Collectively, our findings suggest that chronic mechanical stress contributes to retinal pathology in EL through inflammation-mediated ECM remodeling in RPE cells, thereby advancing the understanding of the interplay among lens dislocation, axial myopia, and retinal degeneration.

## 2. Results

### 2.1. Clinical Characteristics

**Figure 2:**
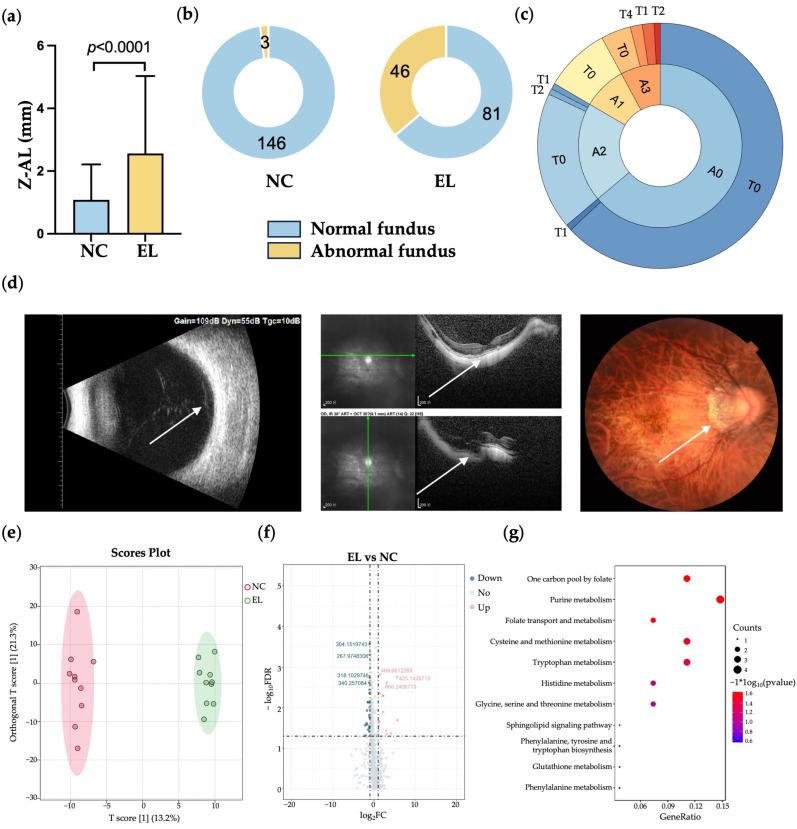
Clinical ocular features of patients with ectopia lentis (EL) compared with healthy controls (NC). (**a**). Z-score adjusted axial length (Z-AL) in NC and EL groups (*p* < 0.0001, unpaired *t*-test). (**b**). Proportion of subjects with retinal abnormalities in each group (*p* < 0.0001, Chi-square test). (**c**). The sunburst chart illustrates the proportional distribution of ATN classifications in EL patients, including the categories of atrophy (A), traction (T), and neovascularization (N). (**d**). Representative images from an EL patient, including B-scan ultrasonography, optical coherence tomography (OCT), and color fundus photography, demonstrate retinal detachment and a tessellated fundus appearance. (**e**). Principal component analysis (PCA) revealed distinct clustering between EL and NC groups, indicating differential metabolic profiles. (**f**). Volcano plot showing significantly upregulated (red) and downregulated (blue) metabolites in EL compared to NC. (**g**). KEGG pathway enrichment analysis of differential metabolites identified upregulated pathways related to one-carbon metabolism, glycine, serine and threonine metabolism, and glutathione metabolism in EL eyes.

A total of 127 patients with EL (EL) carrying FBN1 mutations and 149 healthy controls (NC) were enrolled in this study. The mean age of the EL group was 11.27 (4.00, 14.00) years (range: 0–58 years), significantly younger than the NC group. Within the EL cohort, 24.40% (n = 31) exhibited systemic manifestations, such as skeletal deformities or aortic root dilation. The average axial length (AL) in the NC group was 25.61 ± 1.16 mm, compared to 25.05 ± 3.10 mm in the EL group (Table 1). To account for age-related differences in AL, we calculated a standardized Z-score of AL [15]. The Z-AL in the EL group (2.47 ± 2.56 mm) was significantly higher than that in the NC group (1.09 ± 1.13 mm; *p* < 0.0001), indicating true biometric elongation independent of age (Figure 2a). Retinal abnormalities were observed in 46 of 127 EL patients (36.22%), compared to only 3 of 149 individuals (2.0%) in the NC group. Notably, two EL patients developed RD (Figure 2b). The fundus manifestations of EL patients were classified according to the ATN grading system (A: atrophy, T: tractional, and N: neovascular), with atrophic retinopathy being the most common (Figure 2c) [16].

Figure 2 presents multimodal imaging of a representative EL patient (Figure 2d). In the EL patient, B-scan ultrasonography reveals retinal detachment (RD), OCT imaging displays abnormalities in the RPE layer, and color fundus photography demonstrates significant pigmentary disruption.

### 2.2. Aqueous Humor Metabolomics Profiling

Untargeted metabolomic profiling of aqueous humor revealed significant differences in pathway enrichment between the NC and EL groups. In the EL group, several metabolic pathways were notably upregulated, including folate-mediated one-carbon metabolism, purine metabolism, and glutathione metabolism, indicating enhanced nucleotide biosynthesis and an intensified oxidative stress response. Additionally, altered metabolism of tryptophan, histidine, glycine–serine–threonine, and phenylalanine–tyrosine suggested a remodeling of amino acid metabolism. Notably, sphingolipid signaling was also activated, potentially reflecting changes in membrane dynamics and inflammatory signaling (Figure 2e–g).

### 2.3. Transcriptomic Profiling

**Figure 3:**
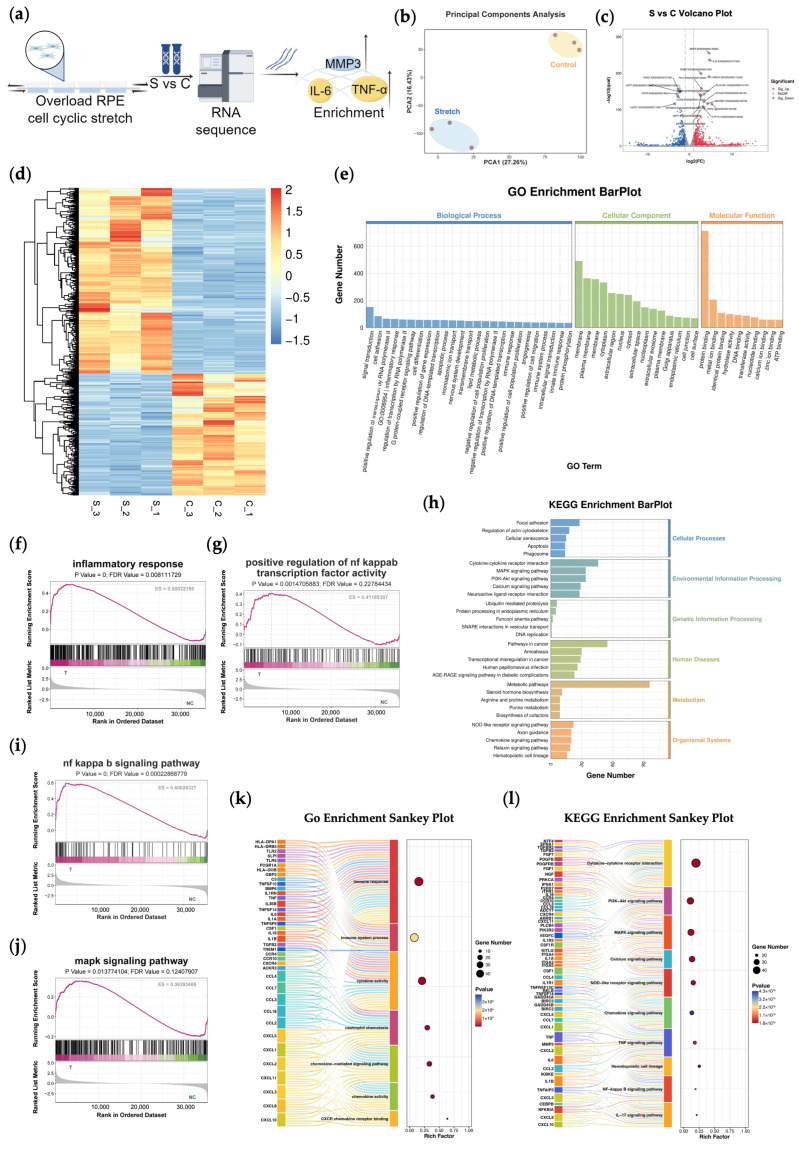
Transcriptomic profiling of ARPE-19 cells under cyclic mechanical stretching. (**a**). Schematic overview of the experimental design, including cyclic stretching application and subsequent RNA sequencing. (**b**). Principal component analysis (PCA) demonstrating transcriptomic separation between stretched (S) and control (C) cells. (**c**). Volcano plot depicting differentially expressed genes (DEGs) between groups. (**d**). Heatmap showing top DEGs based on expression levels. (**e**). Gene Ontology (GO) enrichment bar plot highlighting biological processes, cellular component and molecular function affected by overload stretching. (**f**,**g**). Gene Set Enrichment Analysis (GSEA) revealing significant enrichment of inflammatory response (**f**) and positive regulation of NF-κB transcription factor activity (**g**). (**h**). Kyoto Encyclopedia of Genes and Genomes (KEGG) enrichment bar plot showing key pathways associated with stretching-induced changes. (**i**,**j**). GSEA plots of the NF-κB signaling pathway (**i**) and MAPK signaling pathway (**j**). (**k**,**l**). Sankey plots visualizing enriched GO terms (**k**) and KEGG pathways (**l**) associated with differentially expressed genes.

To explore the molecular consequences of mechanical stress, RNA seq was performed on ARPE-19 cells subjected to overload cyclic mechanical stretching (S, stretching) versus unstretched controls (C, control) (Figure 3a,b). Principal component analysis (PCA) demonstrated clear separation between the two groups. Compared to the control group, a total of 865 genes were significantly upregulated, and 569 genes were significantly downregulated (adjusted *p* < 0.0001, |log_2_FC| ≥ 1). At the transcript level, 2476 transcripts were upregulated and 2060 were downregulated in the stretching group, indicating a broad transcriptional remodeling under mechanical stress (Figure 3c). A heatmap of the top 30 differentially expressed genes further confirmed the transcriptional divergence (Figure 3d). Gene Ontology (GO) enrichment analysis showed that DEGs were significantly enriched in pathways related to inflammatory response, cytokine activity, extracellular region and positive regulation of NF-κB transcription factor activity (Figure 3e). KEGG pathway analysis highlighted the activation of MAPK signaling, and PI3K-Akt signaling (Figure 3h). These data support the hypothesis that mechanical stretching activates inflammation-related gene programs in RPE cells. A Sankey diagram visualized the interconnections between top-ranked GO terms and enriched pathways, underscoring the potential role of mechanotransduction in inflammatory and metabolic responses (Figure 3k,l).

### 2.4. Cell Stretch Assay

**Figure 4:**
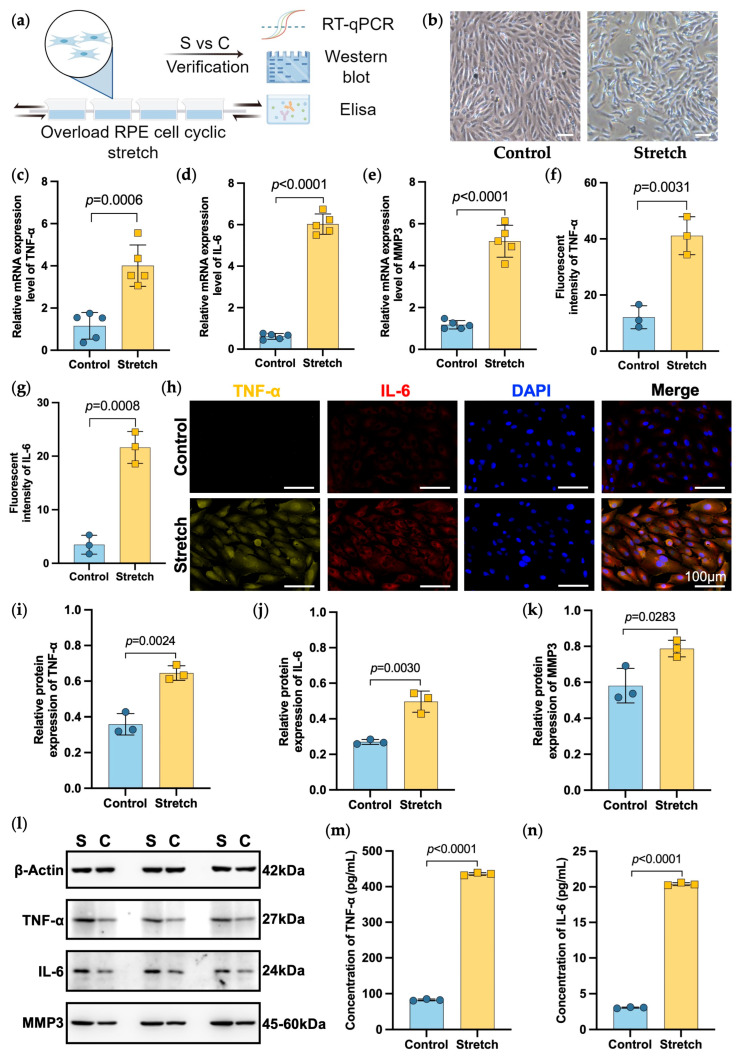
Overload cyclic mechanical stretching induces inflammatory gene and protein expression in ARPE-19 cells. (**a**). Schematic illustration of the experimental workflow: ARPE-19 cells were subjected to overload cyclic mechanical stretching followed by gene and protein expression analyses. (**b**). Representative phase-contrast images showing morphological changes in ARPE-19 cells (scale bar = 100 μm). (**c**–**e**). Real Time-quantitative PCR analysis revealed significant upregulation of TNF-α, IL-6, and MMP3 mRNA expression in stretched cells compared to controls. (**f**–**h**). Quantitative analysis and representative images of TNF-α and IL-6 immunofluorescent staining. (**i**–**k**). Densitometric quantification of Western blot results confirming increased protein expression of TNF-α, IL-6, and MMP3 following mechanical stretching. (**l**). Representative Western blot bands for TNF-α, IL-6, and MMP3 in control and stretched groups. (**m**,**n**). ELISA analysis demonstrated elevated secretion of TNF-α and IL-6 in the culture supernatants of stretched cells. Statistical significance was assessed using unpaired two-tailed *t*-tests. *p* < 0.05 was considered significant.

Following overload cyclic mechanical stretching, ARPE-19 cells exhibited an altered morphology, with more irregular, elongated shapes compared to control cells (Figure 4a,b). To validate RNA-seq findings, we performed RT-qPCR on selected target genes (Figure 4c–e). Consistent with sequencing results, expression of TNF-α, IL-6 and MMP3 was significantly elevated in the stretching group compared to controls (*p* = 0.0006, *p* < 0.0001 and *p* < 0.0001, respectively). IF staining of TNF-α and IL-6 showed increased expression in the stretching group. Quantitative analysis further revealed that TNF-α expression was significantly elevated (*p* = 0.0031), and IL-6 levels were also markedly increased (*p* = 0.0008) in the stretching group (Figure 4f–h). Consistently, WB analysis confirmed increased protein expression of TNF-α, IL-6 and MMP3 (*p* = 0.0024, *p* = 0.0030 and *p* = 0.0283, respectively) (Figure 4i–k). ELISA of the culture supernatants also revealed significantly higher secreted levels of both inflammatory mediators (TNF-α and IL-6) in the stretching group, corroborating a mechanosensitive inflammatory phenotype in RPE cells (*p* < 0.0001 and *p* < 0.0001, respectively) (Figure 4l–n).

## 3. Discussion

Clinical observations have shown that patients with EL exhibit pronounced axial elongation and a significantly higher incidence of vitreoretinal complications, including peripheral retinal degeneration, posterior staphyloma, and retinal detachment—which often result in severe visual impairment beginning in adolescence [3,16,17,18]. Untargeted metabolomic profiling of aqueous humor from EL patients revealed a significant enrichment of proinflammatory metabolites, including prostaglandin E2 (PGE2), hyaluronic acid, and lactate, suggesting the presence of a sustained proinflammatory intraocular environment [19,20,21]. To explore the potential mechanism linking axial elongation to inflammation and retinal pathology, we established an in vitro mechanical stretching model to mimic the biomechanical stress associated with excessive axial growth [2]. Cyclic mechanical stretching applied to ARPE-19 cells induced robust activation of inflammatory and extracellular matrix (ECM) remodeling pathways, with significant upregulation of TNF-α, IL-6 and MMP3. These results support a mechanobiological cascade in EL pathogenesis, wherein progressive axial elongation induces mechanical stress on RPE cells, subsequently triggering inflammatory responses and ECM remodeling, ultimately leading to retinal structural abnormalities [11,13,22,23].

Genetically, the majority of EL patients harbor mutations in the FBN1 gene, which encodes fibrillin-1—a key ECM component essential for maintaining the biomechanical integrity of the ocular wall [24,25]. Functional studies have elucidated two main pathogenic mechanisms of FBN1 mutations: (1) haploinsufficiency (HI), which impairs TGF-β sequestration and disrupts microfibril assembly, and (2) dominant-negative (DN) mutations, which result in misfolded fibrillin-1 proteins that compromise ECM structural stability [26]. Both types of mutations can impair the biomechanical properties of the scleral tissue, leading to ECM disintegration and decreased scleral rigidity, thereby promoting progressive axial elongation [27,28]. Our previous investigations and clinical observations have further demonstrated that AL extension in EL patients is more closely associated with these underlying FBN1 mutations than with lens dislocation alone, as carriers exhibit a significantly higher predisposition to excessive axial elongation and associated retinal abnormalities [16]. Consequently, FBN1-driven axial elongation imposes chronic mechanical strain on the posterior segment, establishing the biological rationale for using RPE cells stretching models to investigate secondary retinal remodeling and inflammatory activation.

In this study, cyclic mechanical stretching of RPE cells led to a marked upregulation of multiple proinflammatory cytokines, most notably TNF-α and IL-6, along with activation of the NF-κB and MAPK signaling pathways. These results suggest that RPE cells can directly sense and respond to mechanical stress associated with axial elongation by producing inflammatory mediators. The upregulation of these factors was confirmed at both the mRNA and protein levels, underscoring the mechanosensitive nature of RPE cells and their role in sustaining a proinflammatory intraocular environment. This mechanotransductive response may offer a molecular explanation for the chronic, low-grade inflammation observed in patients with EL.

Among the cytokines identified, IL-6 is known to promote ECM degradation and retinal injury under stress conditions [29]. TNF-α, another key mediator, regulates MMPs expression under mechanical load. Notably, MMP3, a matrix metalloproteinase, plays a central role in ECM degradation and may disrupt the blood–retinal barrier and compromise the structural integrity of neural tissues [14,29]. In our study, mechanical stretching robustly induced MMP3 expression and secretion in RPE cells, suggesting that mechanical stress not only induces inflammation but also triggers ECM remodeling. This process may contribute to structural weakening of the RPE and underlying tissue, potentially driving the formation of posterior staphyloma, retinal degeneration, and RD—serious complications frequently observed in EL patients. Taken together, our findings suggest that chronic mechanical stress associated with axial elongation acts as a pathological stimulus that activates inflammatory and ECM-remodeling pathways in RPE cells. The marked upregulation of TNF-α, IL-6, and MMP3 points to downstream pathways associated with retinal degeneration and tissue disorganization. Moreover, the elevated levels of TNF-α, IL-6 and MMP3 may serve as potential biomarkers and therapeutic targets for early retinal injury in EL.

Several limitations should be acknowledged. The applied mechanical stretching was isotropic and uniform, which does not fully capture the complex anisotropic forces present in vivo, particularly in eyes with posterior staphyloma or asymmetrical globe expansion seen in EL patients. The age difference between groups also represents a potential confounder, as axial length progression is age-dependent; thus, the observed metabolic differences should be interpreted cautiously as associative rather than causal. Moreover, our study lacks in vivo histological or functional validation using animal models. Currently, there is a lack of genetic animal models that accurately mimic the pathological processes observed in EL patients, which limits the ability to obtain comprehensive evaluation results for future clinical translation studies [30]. Pharmacologic inhibition of IL6 or MMP3 pathways in stretched RPE cells could assess the therapeutic efficacy of targeting mechanosensitive inflammatory pathways. Future investigations should include validation using primary RPE cells or retinal organoids that better replicate native physiology.

## 4. Materials and Methods

### 4.1. Study Subjects

This study enrolled patients with EL carrying FBN1 mutations who were diagnosed and treated at the Eye and ENT Hospital of Fudan University, Shanghai, China. Healthy individuals who underwent implantable collamer lens (ICL) implantation at the same institution served as the healthy control group. Comprehensive ophthalmic examinations included slit-lamp biomicroscopy, B-scan ultrasound, fundus photography (CLARUS 500, Carl Zeiss Meditec AG, Jena, Germany), and optical coherence tomography (Spectralis OCT, Heidelberg Engineering, Heidelberg, Germany, or Cirrus OCT, Carl Zeiss Meditec, Dublin, CA, USA). AL was measured using the IOLMaster 700 (Carl Zeiss Meditec, Jena, Germany) and a rotating Scheimpflug camera (Pentacam, Oculus Optikgeräte GmbH, Wetzlar, Germany). To adjust for age-related variation in AL, Z-scores were calculated as Z-AL = (measured AL − normative AL)/normative standard deviation (SD) [15]. The study was conducted in accordance with the Declaration of Helsinki and approved by the Ethics Committee (ChiCTR2000039132) of Eye and ENT Hospital of Fudan University. Written informed consent was obtained from all participants or their guardians in subjects under 18 years old.

### 4.2. Aqueous Humor Metabolomics

Aqueous humor samples were collected from 10 EL patients during cataract surgery and from 10 healthy controls undergoing ICL implantation. Samples were immediately frozen and stored at −80 °C. Untargeted metabolomic profiling was performed using liquid chromatography–mass spectrometry (LC-MS) by Biotech (Shanghai, China). Metabolites were identified and quantified using MetaboAnalyst 5.0.

### 4.3. Cell Culture

Human retinal pigment epithelial cells (ARPE-19; ATCC, Manassas, VA, USA) were cultured in αMEM medium (Gibco, Thermo Fisher Scientific, Waltham, MA, USA) supplemented with 10% fetal bovine serum (FBS; Gibco) and 1% penicillin–streptomycin. Cells were maintained at 37 °C in a humidified incubator with 5% CO_2_ and were used at 80–90% confluence.

### 4.4. Cyclic Mechanical Stretch Assay

ARPE-19 cells were seeded onto flexible-bottomed culture plates precoated with rat tail collagen (Sigma-Aldrich, St. Louis, MO, USA). Overloaded cyclic mechanical stretching (20% elongation, 1 Hz, sine waveform, 2 h/day) was applied using the cell-tank system (Hangzhou CellMax Surface Technology Co., Ltd., Hangzhou, China). Control cells were maintained under identical conditions without mechanical loading.

### 4.5. Real Time-Quantitative PCR (RT-qPCR)

Total RNA was extracted from ARPE-19 cells using the EZ-press RNA Purification Kit (EZBioscience, San Diego, CA, USA). RNA concentration and purity were assessed using a NanoDrop ND-2000 spectrophotometer (Thermo Fisher Scientific, Waltham, MA, USA); only samples with an A260/A280 ratio between 1.9 and 2.1 were included. All RNA samples were treated with DNase I (Thermo Fisher Scientific, Waltham, MA, USA) to remove residual genomic DNA. cDNA was synthesized using 1 μg of total RNA and the 4× Reverse Transcription Master Mix (EZBioscience, San Diego, CA, USA). The resulting cDNA was diluted 1:5, and 2 μL (equivalent to 40 ng starting RNA) was used per qPCR reaction. qPCR was performed using 2× SYBR Green qPCR Master Mix (EZBioscience, San Diego, CA, USA) on a LightCycler 480 II system (Roche, Basel, Switzerland). Each condition was analyzed in triplicate with three independent biological replicates. GAPDH was used as an internal control, and relative gene expression was calculated using the 2^–ΔΔCt^ method. Samples with Ct values above 35 were excluded as below the detection limit. Primer sequences for TNF-α, IL-6, MMP3, and GAPDH are listed in Supplementary Table S1.

### 4.6. RNA Sequencing (RNA-Seq)

ARPE-19 cells were harvested on day 4 following either cyclic mechanical stretching or control conditions for RNA-Seq and data analysis. Total RNA was extracted using TRIzol (Invitrogen, Carlsbad, CA, USA), and mRNA was purified with Dynabeads Oligo (dT). RNA integrity was verified. cDNA libraries with average insert sizes of 300 ± 50 bp were generated and sequenced using 2 × 150 bp paired-end reads on the Illumina NovaSeq 6000 platform (Illumina, San Diego, CA, USA). Raw reads were aligned to the human reference genome hg38 (GRCh38) using STAR v2.7.10a (two-pass mode). Gene-level counts were obtained with featureCounts (Subread v2.0.3) using the GENCODE v41 annotation. Differential expression analysis between stretching and control groups was performed with DESeq2 v1.36.0 in R v4.2.0, considering genes with an adjusted *p*-value < 0.05 (Benjamini-Hochberg) and |log_2_fold change| ≥ 1 as significant. All bioinformatic analyses were conducted using OmicStudio tools (https://www.omicstudio.cn/tool) accessed on 5 May 2025. Experiments were performed in triplicate.

### 4.7. Immunofluorescence Staining (IF)

For both the control and stretching groups, the medium was removed, and the cells were washed three times with phosphate-buffered saline (PBS). Cells were then fixed in 4% paraformaldehyde for 30 min at room temperature. Following fixation, cells were permeabilized with 0.5% Triton X-100 for 30 min and blocked with 5% bovine serum albumin for 30 min. Primary antibodies against TNF-α (Gibco, Cat# 300-01A-50UG) or IL-6 (Thermo Fisher Scientific, M620) were applied and incubated overnight at 4 ◦C. The following day, the cells were washed three times with PBS and incubated with secondary antibodies for 2 h. The secondary antibodies used were Alexa Fluor^®^ 568-conjugated goat anti-rabbit IgG (H + L) (Abcam, Cambridge, UK, Cat# ab175473) and Alexa Fluor^®^ 647-conjugated goat anti-mouse IgG (H + L) (Abcam, Cat# ab150115). Nuclei were counterstained with DAPI (Beyotime, Shanghai, China, Cat# C1002; 1 μg/mL) for 5 min. Samples were imaged using a wide-field fluorescence microscope (DMi8, Leica Microsystems, Wetzlar, Germany).

### 4.8. Western Blotting (WB)

Total protein was extracted from RPE cells using RIPA lysis buffer (Solarbio, Beijing, China) supplemented with protease and phosphatase inhibitors (Solarbio, Beijing, China). Protein concentrations were measured using a BCA Protein Assay Kit (Solarbio, Beijing, China). Equal amounts of protein (20–30 µg) were separated by SDS-PAGE and transferred to PVDF membranes. Membranes were blocked with 5% BSA for 1 h at room temperature and incubated overnight at 4 °C with primary antibodies: TNF-α (Gibco, Cat# 300-01A-50UG), IL-6 (Thermo Fisher Scientific, M620), MMP3 (Thermo Fisher Scientific, Cat# MA5-17123), and GAPDH (Thermo Fisher Scientific, Cat# PA1-987). The next day, membranes were incubated for 1 h at room temperature with HRP-conjugated secondary antibodies: goat anti-mouse IgG (H + L) (Jackson ImmunoResearch, Cat# 115-035-003) or goat anti-rabbit IgG (H + L) (Jackson ImmunoResearch, Cat# 111-035-003). Protein bands were visualized using enhanced chemiluminescence (Thermo Fisher Scientific) and imaged with a chemiluminescence system (Tanon 5200, Shanghai, China).

### 4.9. Enzyme-Linked Immunosorbent Assay (ELISA)

Culture supernatants from ARPE-19 cells under different stretching conditions were collected and centrifuged to remove debris. Human TNF-α and IL-6 levels were quantified using ELISA kits (eBioscience, Cat#14-7069 for IL-6 and Thermo Fisher Scientific, Cat# MA5-17123 for MMP3), following the manufacturer’s protocols. Absorbance was measured at 450 nm using a microplate reader (BioTek, Winooski, VT, USA), and concentrations were calculated using standard curves.

### 4.10. Statistical Analysis

Statistical analyses were performed using SPSS (version 20.0; IBM Corp., Winooski, NY, USA) and GraphPad Prism v9.0 (GraphPad Software, San Diego, CA, USA). Quantitative data are presented as mean ± standard deviation (SD). Group comparisons were conducted using Student’s t-test, Mann–Whitney U test, or chi-squared test, as appropriate. *p*-value < 0.05 was considered statistically significant.

## 5. Conclusions

In conclusion, this study provides mechanistic evidence linking axial elongation in EL to RPE-driven inflammation and ECM remodeling. Our findings highlight the necessity of early clinical intervention in patients with EL to mitigate vision-threatening complications. When combined with close retinal surveillance for the early detection of vitreoretinal abnormalities, these strategies offer a more comprehensive approach to managing secondary retinal changes associated with EL. Ultimately, these insights may inform the development of future therapeutic strategies targeting mechanosensitive inflammatory pathways in the posterior segment.

## Figures and Tables

**Table 1:** Clinical characteristics of Ectopia Lentis (EL) patients and healthy controls (NC).

Variable	NC	EL	P
Num	149	127	
Age (years)	22 (19.36, 22)	7 (4.00, 14.00)	<0.001
Male	93 (62.42%)	78 (61.40%)	0.865
Family history	0	53 (41.70%)	<0.001
Ectopia lentis degree			
Mild	/	12 (9.40%)	/
Moderate	/	69 (54.30%)	/
Severe	/	46 (36.20%)	/
Systemic diseases	0 (0.00%)	31 (24.40%)	
Uncorrected visual acuity (logMAR)	1.35 ± 0.25	0.99 ± 0.36	<0.001
Best-corrected visual acuity (logMAR)	0.05 ± 0.08	0.60 ± 0.34	<0.001
Intraocular pressure (mmHg)	15.42 ± 2.65	14.44 ± 3.46	0.012
Axial length (mm)	25.61 ± 1.16	25.05 ± 3.10	0.045
Z-AL	1.08 ± 1.12	2.56 ± 2.47	<0.001
Anterior chamber depth (mm)	3.70 ± 0.25	3.21 ± 0.51	<0.001
White to white (mm)	12.19 ± 0.41	12.02 ± 0.63	0.018
Lens thickness (mm)	3.50 ± 0.20	3.83 ± 0.95	<0.001
Mean keratometry (D)	42.56 ± 1.50	39.81 ± 1.77	<0.001
Pupil diameter (mm)	3.98 ± 0.50	4.28 ± 1.67	0.042

## Data Availability

All data, models, or code generated or used during the study are available from the corresponding author by request.

## Supplementary Materials

The following supporting information can be downloaded at https://www.mdpi.com/article/10.3390/ijms27114870/s1.

## Abbreviations

RPE	Retinal pigment epithelial
ECM	extracellular matrix
EL	ectopia lentis
AL	axial length
Z-AL	Z-score-adjusted axial length
ATN	A: atrophy, T: tractional, and N: neovascular
RT-qPCR	Real Time-quantitative PCR
IF	immunofluorescence staining
WB	Western blotting
ELISA	enzyme-linked immunosorbent assay
RD	retinal detachment
ICL	implantable collamer lens
FBS	fetal bovine serum
PBS	phosphate-buffered saline
SD	standard deviation
NC	healthy control
FAK	focal adhesion kinase
TNF-α	tumor necrosis factor-α
IL-6	interleukin-6
MMP3	matrix metalloproteinase-3
OCT	optical coherence tomography
PCA	Principal component analysis
GO	Gene Ontology
DEGs	differentially expressed genes
KEGG	Kyoto Encyclopedia of Genes and Genomes
PGE2	prostaglandin E2
NF-κB	Nuclear Factor kappa-light-chain-enhancer of activated B cells
MAPK	Mitogen-Activated Protein Kinase
PI3K-Akt	Phosphoinositide 3-kinase/Protein Kinase B
GSEA	Gene Set Enrichment Analysis
HI	haploinsufficiency
DN	dominant-negative
