# Supplementary material for: Excessive Stretching Drives RPE Inflammation and ECM Remodeling in Ectopia Lentis Retinopathy
Source: Int J Mol Sci. 2026 May 28;27(11):4870. doi: 10.3390/ijms27114870 (PMC13257021; doi:10.3390/ijms27114870)
Supplement: Supplementary file 1 [file ijms-27-04870-s001.zip › ijms-4181754-supplementary.pdf]

Table S1. Primer sequences used for RT-qPCR.

	Primer Direction	Sequence (5′-3′)	Source
TNF-α	Forward	CCTCTCTCTAATCAGCCCTCTG	OriGene (NM_000594)
	Reverse	GAGGACCTGGGAGTAGATGAG	OriGene (NM_000594)
IL-6	Forward	ACTCACCTCTTCAGAACGAATTG	OriGene (NM_000600)
	Reverse	CCATCTTTGGAAGGTTCAGGTTG	OriGene (NM_000600)
MMP3	Forward	CTGGACTCCGACACTCTGGA	OriGene (NM_002422)
	Reverse	CAGGAAAGGTTCTGAAGTGACC	OriGene (NM_002422)
GAPDH	Forward	GGAGCGAGATCCCTCCAAAAT	OriGene (NM_001256799)
	Reverse	GGCTGTTGTCATACTTCTCATGG	OriGene (NM_001256799)
